# A new algorithm to extract hidden rules of gastric cancer data based on ontology

**DOI:** 10.1186/s40064-016-1943-9

**Published:** 2016-03-10

**Authors:** Seyed Abbas Mahmoodi, Kamal Mirzaie, Seyed Mostafa Mahmoudi

**Affiliations:** Department of Computer Engineering, Yazd Science and Research Branch, Islamic Azad University, Yazd, Iran; Department of Computer Engineering, Maybod Branch, Islamic Azad University, Maybod, Iran; Department of Oral Pathology, Birjand University of Medical Sciences, Birjand, Iran

**Keywords:** Gastric cancer, Ontology, Data mining, Apriori

## Abstract

Cancer is the leading cause of death in economically developed countries and the second leading cause of death in developing countries. Gastric cancers are among the most devastating and incurable forms of cancer and their treatment may be excessively complex and costly. Data mining, a technology that is used to produce analytically useful information, has been employed successfully with medical data. Although the use of traditional data mining techniques such as association rules helps to extract knowledge from large data sets, sometimes the results obtained from a data set are so large that it is a major problem. In fact, one of the disadvantages of this technique is a lot of nonsense and redundant rules due to the lack of attention to the concept and meaning of items or the samples. This paper presents a new method to discover association rules using ontology to solve the expressed problems. This paper reports a data mining based on ontology on a medical database containing clinical data on patients referring to the Imam Reza Hospital at Tabriz. The data set used in this paper is gathered from 490 random visitors to the Imam Reza Hospital at Tabriz, who had been suspicions of having gastric cancer. The proposed data mining algorithm based on ontology makes rules more intuitive, appealing and understandable, eliminates waste and useless rules, and as a minor result, significantly reduces Apriori algorithm running time. The experimental results confirm the efficiency and advantages of this algorithm.

## Introduction

Large data sets are idle in database of companies, universities, etc. Using the hidden information in these data bases is based on the efficient management. Data mining is looking for hidden relationships in databases. This process is more than just a simple retrieving data and lets researchers to find new information from data (Alizadehsani et al. 2013).

Cancer is the leading cause of death in economically developed countries and the second leading cause of death in developing countries (Jemal et al. 2011). Gastric cancer is the fourth-most common cancer (Price et al. 2012) and the second leading cause of cancer deaths in the worldwide (Ferlay et al. 2010). According to the latest researches conducted in Iran in 2008, 9.3 % of the common cancer is related to stomach cancer and it is the third most common cancer among men and women in the country (Etemad et al. 2012). It is necessary to further examine the factors affecting the incidence of the disease Due to the prevalence of the disease and the high mortality rate of gastric cancer.

Today, the medical knowledge of the data is very extensive on the symptoms of patients with various diseases and the ways to help them with the diagnosis of these diseases. Analyzing and considering all the engaged factors by one person are usually difficult. Thus, the need for an automated system to help discover the rules and patterns and predict future events is fully felt. Data mining, as the provider of this automated system, helps many medical advances, especially in the field of diagnosis of various diseases and obtaining useful relations among factors available in the data (Chou et al. 2004).

Kirshners et al. (2012) used a data set including 819 samples (24 positive and 795 negative samples) and 31 features and three algorithms CN2 Rules and C4.5 and Naive Bayes to diagnose stomach cancer. The results showed that sex and protein HER-1, are important factors in the diagnosis and classification of gastric cancer. Experimental results showed the average sensitivity >50 and 86–100 % at most, at the same time—having classification accuracy and specificity close to 65–70 %. Silvera et al. (2014) used classification tree analysis to analyze data from a population-based case–control study (1095 cases, 687 controls) conducted in Connecticut, New Jersey, and Western Washington State. Frequency of reported gastroesophageal reflux disease symptoms was the most important risk stratification factor for esophageal adenocarcinoma, gastric cardia, and noncardia gastric, with dietary factors (esophageal adenocarcinoma, noncardia gastric), smoking (esophageal adenocarcinoma, gastric cardia), wine intake (gastric cardia, noncardia gastric), age (noncardia gastric), and income (noncardia gastric) appearing to modify the risk of these cancer sites. For esophageal squamous cell carcinoma, smoking was the most important risk stratification factor, with gastroesophageal reflux disease, income, race, noncitrus fruit, and energy intakes further modifying risk. Wang et al. (2012) used hierarchical clustering on 14 available clinical factors from three categories, i.e., the clinical background, immunohistochemistry data, and the caner’s stage information. The results showed that that two clinical factors, Her-1 and gender, can clearly characterize and differentiate these three groups.

In classifying and clustering somehow these methods derive patterns from the data set. For example, in the clustering, a pattern of similarity is defined and assessed. But the main focus is on discovering the hidden factors and influencing factors in this dangerous disease by the help of discovering the association rules that no research evidence has been carried out in this regard. Most conducted researches that use discovery of association rules are applied for data collection of other cancers.

Since primary prevention, by control of modifiable risk factors and increased surveillance of persons at increased risk, is important in decreasing morbidity and mortality of this harmful disease (Bathaie and Mohagheghi 2012), in this study, the effect of some new features which was not considered in previous studies are investigated. Although the use of traditional data mining techniques such as association rules helps to extract knowledge from large data sets, sometimes the results obtained from a data set are so large that it is a major problem. In fact, one of the disadvantages of this technique is a lot of nonsense and redundant rules due to the lack of attention to the concept and meaning of items or the samples. This paper presents a new method to discover association rules using ontology to solve the expressed problems. Experiments using the proposed algorithm have given results that are more concise and precise than the results obtained using Traditional data mining techniques. The quantitative comparison was performed according to the number of pruned rules. The qualitative comparison was performed by human evaluation.

## Background

Data mining is the process of extracting hidden knowledge from data. It can reveal the patterns and relationships among large amount of data in a single or several data sets. Data mining is used in various applications such as crime detection, risk evaluation market analysis, etc. Several industries like banking, insurance, and marketing use data mining to reduce costs, and increase profits (Witten et al. 2005).The data mining process uses several techniques from statistics and artificial intelligence in a variety of activities or application areas. The main activities are as follows (Goebel and Gruenwald 1999):

- *Association* The identification of relationships between items in which the presence of one pattern implies the presence of another pattern; e.g., most patients who receive prescriptions for medication “A” also receive prescriptions for medication “B”.
- *Classification* Classification involves identifying profiles of classes in terms of their attributes and determining which of these predefined classes a new item belongs to. For example, given particular classes of patients with different medical treatment responses, the classification is used to identify the form of treatment to which a new patient is most likely to respond.
- *Clustering* Clustering is used to identify a set of classes in which particular items are grouped according to their characteristics. Clustering is best used to identify groups of items that are similar. For example, based on a patient data set, clustering can be used to identify subgroups of patients with similar treatment schemas.
- *Regression* For a set of items, regression is the analysis of the relationships of dependence between the values of the attributes. A model is automatically produced that can predict attribute values for new items. For example, with a data set for the medical procedures used in a treatment schema, regression is used to build a model that can predict the sequence of procedures in the treatment.
- *Prediction* For a specific item and a corresponding model, the capacity for prediction is the ability to predict the value of a specific attribute. For example, in a predictive model for treatment schema, prediction is used to determine the next procedure in the sequence of treatment.

### Association rule mining

The use of association rules is a popular technique of mining data; the technique shows the correlation between sets of items in a series of data or transactions. A rule for a given dataset has the form A → B, where A and B are conditions on the values of the features. For each rule, two terms, namely support and confidence, are defined. Support of a rule means the proportion of data which satisfies both the left hand and right hand sides of that rule. Confidence means the probability of finding the right hand side of the rule in those item sets which satisfy the left hand side conditions as shown below:

1 $$ {\text{Confidence}}\, ( {\text{A}} \to {\text{B)}} = \Pr ({\text{A}} \cup {\text{B}})/\Pr ({\text{A}}) $$

To obtain rules, firstly conditions on features that have the highest probabilities are derived from the dataset. Then these conditions are split in all possible ways to form smaller conditions A and B. The most popular algorithm for obtaining association rules is Agrawal’s apriori (Agrawal and Srikant 1994). Considering a fixed confidence value, the setting of the support threshold will determine whether too many association rules are set or important relationships in the data are missed.

### Ontology

According to Gruber (1993), ontology is a conceptualization of a specification. Instead of using this very broad explanation, the present research takes a more tangible definition of ontology as a domain representation in which the concepts and the relations among them become explicit to allow either human negotiation of the denotations and/or machine inferences for a specific application. People build domain ontology, and consequently, rarely is there a consensual understanding of all concepts, and different ontologies can be built to describe the same domain. Since the 1990s, There are many studies not only on techniques with which to build (Musen 1994) and map ontologies (Euzenat and Shvaiko 2007) but also on applications that could benefit from representing knowledge.

Using domain ontology, such as knowledge-based systems, information retrieval mechanisms, agent communication language definition and search methods. The present research investigates the effects of using domain ontology to improve the precision and recall of association-rule extraction from large databases.

Identifying the concepts and relations between concepts is crucial to building an effective domain ontology. Although the semantics of the relations can be defined as one builds an ontology, there are some relations with established meanings, such as “is-a”, “part-of” and “attribute-of”. The first relation, is-a, comes from the set theory relation set and subset. Consequently, the subset carries all definitions of the set. If Peter is-a human, then Peter inherits all characteristics of being a human. This description can be very useful in describing concepts in a more concise way.

The second relation, part-of, brings to bear the concept of composition/decomposition. The parts make the whole. A motor engine and chassis are parts of a car. A motor alone cannot be considered a car.

Attribute-of is a simple relation that represents properties of the concepts. For example, a car has a color and might have an owner. Both are attributes of a car. It is natural to map the way one describes a domain in natural language and the relations the concepts present. However, care needs to be taken for language might be misleading. A car has a color and has a motor. Although, in general, we use the same verb to connect the car and color, and the car and motor engine, the relations between these concepts are different. In the first one, the relation is clearly about characteristics of the object, while in the second one, the relation refers to composition.

## The medical data set

The S-Abbas Mahmoodi dataset contains the records of 470 patients, each of which have 29 features. All features can be considered as indicators of gastric cancer for a patient, according to medical literature. However, some of them have never been used in data mining based approaches for gastric cancer diagnosis. The features are arranged in four groups: personal characteristics and behavior and systemic features and the stomach. Table 1 presents the features of S-Abbas Mahmoodi dataset along with their valid ranges, respectively.

**Table 1:** Features of S-Abbas Mahmoodi dataset

Feature type	Feature name	Range
Personal characteristics and behavior	Sex	Male, female
	Blood group	A, B, AB, O
	Smoking	Yes, no
	Alcohol consumption	Yes, no
	Exposed to chemicals	Yes, no
	BMI	BMI >30, 25 < BMI > 29.5, 18.5 < BMI > 24.9, BMI <18.5
	Motility	Light, medium, high
	Age	40>, 41 <> 60, 61<
	Salt consumption	Not eat, high low
	Consumption of vegetable	Daily, 1–3 times a week, 1–3 times a month
	Consumption of smoked food	Not eat, 1–3 times a week, 1–3 times a month
	Milk consumption	Yes, no
	Fast food consumption	Not eat, 1–3 times a week, 1–3 times a month
	Consumption of fried foods	Not eat, 1–3 times a week, 1–3 times a month
	Fruit consumption	Daily, 1–3 times a week, 1–3 times a month
	Food storage container	Aluminum, plastic, Chinese, style, copper
	Dish cooking	Aluminum, Teflon, copper
Systemic features and the stomach	History of allergy	Yes, no
	Family history of cancer	Yes, no
	Family history of gastric cancer	Yes, no
	History of cardiovascular disease	Yes, no
	Gastric cancer	Yes, no
	General status of cancer	Good, so–so, poor
	History of gastric reflux	Yes, no
	history of stomach surgery	Yes, no
	History of gastritis	Yes, no
	History of stomach infection	Yes, no
	Mucosa status	Normal, swollen, red, sore
	Cancer site	Cardia, non cardia

## Methods

### Gastric cancer ontology

Many languages can be used to represent ontologies, from highly informal to rigorously formal (Eccher et al. 2013). The desired Ontology is implemented by using MATLAB software. Precise definition of concepts on cancer, especially gastric cancer, diagnostic methods, therapy methods, etc., are a necessity. For this purpose, regular meetings were held between computer professionals and gastric cancer specialists.

**Fig. 1:**
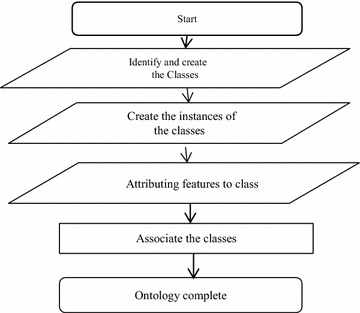
Ontology design process

The design of Ontology was conducted by using the obtained information, the discussions and the framework presented in Fig. 1. The design process of Ontology is shown in Fig. 1. As seen in the figure, the first step is identifying and creating classes. In this step, after identifying the discussed scope as well as investigating and understanding the discussed concepts, domain classes are identified. The second step is determining attributes available in the database after pre-processing. The class of the available attributes in database is determined in the third step. Relations between classes are identified according to domain knowledge and understanding the concepts in the domain. Now Ontology is completed.

**Fig. 2:**
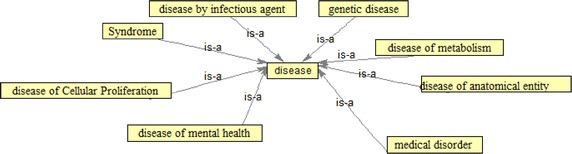
Disease ontology

For the design and implementation of Ontology, high level concepts of the desired domain of the disease, namely disease, are considered first. High level Ontology of disease and its kinds are shown in Fig. 2.

In general, diseases are divided into eight main categories: genetic disease, syndrome, medical disease, disease of metabolism, disease of cellular proliferation, disease by infectious agent, disease of anatomical entity and disease of mental health. In fact, nine general concepts of diseases were determined. All the eight main categories of disease are a subclass of the main disease class linked to its main class by IS–A relationship. Disease ontology is shown in Fig. 2.

**Fig. 3:**
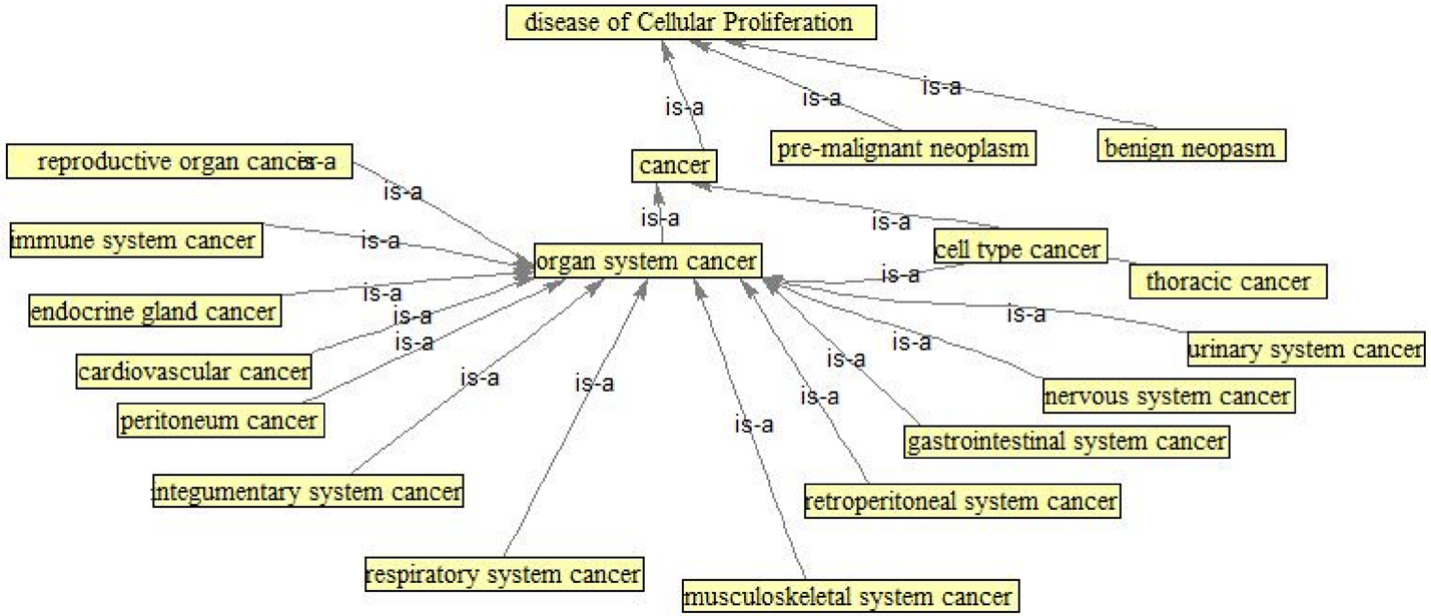
Cancer ontology

Ontology shows information about cancers including three main classes such as disease of cellular proliferation, cancer and organ system cancer. Of course, cancer class of body systems is a subclass of cancer class and cancer class is a subclass of the main classes of cell proliferation diseases. Cancer ontology is shown in Fig. 3.

Since the desired disease in this study is gastric cancer, gastric cancer ontology is designed and investigated in the following. It should be mentioned that all relationships between classes and subclasses are self-defined.

**Fig. 4:**
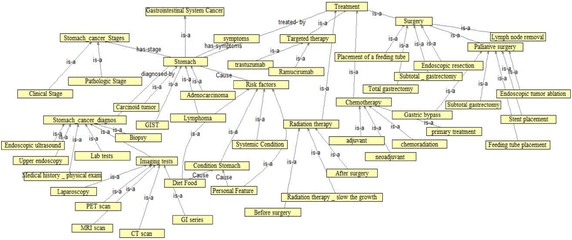
Gastric cancer ontology

Figure 4 shows ontology including domain knowledge and a set of concepts, classes and subclass, and relationships between classes. Main concepts on gastric cancer include some main sections such as types of stomach cancers, treatment, risk factor, symptom, clinical stage, and diagnose. In fact, each of these sections is main classes of ontology including different classes and subclasses.

Classes and relationships: each class with its subclasses is introduced in the following. Gastric cancer includes four subclasses adenocarcinoma, lymphoma, gastrointestinal (GI) stromal tumor carcinoid tumor. Treatment class includes four subclasses surgery, chemotherapy, targeted therapy and radiation therapy which each one includes some subclasses. Surgery class includes six subclasses endoscopic resection, subtotal gastrectomy, total gastrectomy, placement of a feeding tube, lymph node removal and palliative surgery for unresectable cancer which palliative surgery consists of four subclasses. Chemotherapy subclass includes four subclasses neoadjuvant, adjuvant, primary treatment and chemoradiatin. Targeted therapy consists of two subclasses, trastuzumab and ramucirumab. Radiotherapy includes three subclasses before surgery, after surgery and Radiation therapy can be used to slow the growth. Class of cancer stage: cancer stage includes two subclasses clinical stage and pathologic stage. Diagnose class: this class includes six subclasses medical history and physical exam, upper endoscopy, endoscopic ultrasound, biopsy, imaging tests and lab test. Image test consists of five subclasses upper GI series, computed tomography scan, magnetic resonance imaging (MRI) scan, positron emission tomography (PET) scan and laparoscopy.

#### Ontology of the risk factors of gastric cancer

As mentioned, ontologies of disease, cancer, and gastric cancer were considered for demonstrating current concepts related to the whole domain. Given to the extent and complexity of the ontology of gastric cancer in this study as well as the examined attributes on the risk factors of gastric cancer and the effective factors in the incidence of this disease, otology of the risk factors of gastric cancer which is, in fact, a part of the main ontology of gastric cancer are taken into consideration.

**Fig. 5:**
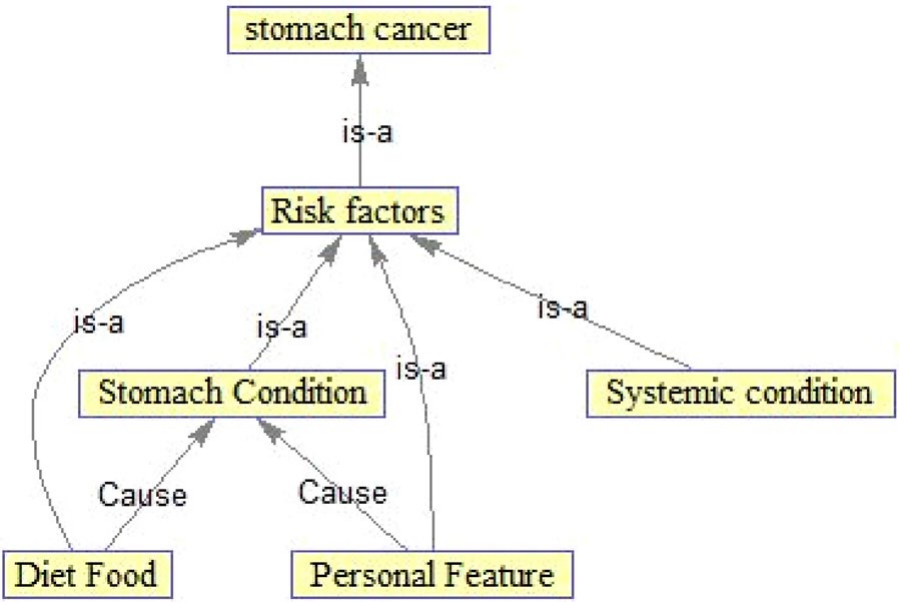
Ontology of the risk factors of gastric cancer

Figure 5 shows the risk factors ontology of gastric cancer. The risk factor class is the main class of this ontology and also one of the main classes of gastric cancer ontology which will be discusses in details because of its significant importance.

Risk factors are something that influences the chance of catching a disease such as cancer (Reyes-Ortiz et al. 2013) Different cancers have various risk factors. Some of risk factors are changeable like cigarette smoking, but some other ones are not changeable like family history. Having a factor does not mean that a person has certainly cancer. Perhaps there are many people having cancer but they have either a few risk factors or no risk factors. Scientists have stated several risk factors in catching the disease of gastric cancer. According to the data set and also with the help of a pathologist, risk factors of gastric cancer are divided into four categories including stomach condition, personal feature, systemic condition, and diet food.

As seen in Fig. 5, each of these categories is a subclass of the main class. Two relationships are used in this ontology including IS–A in the hierarchical relationships and cause in the non-hierarchical relationships.

#### Mapping database to ontology

**Fig. 6:**
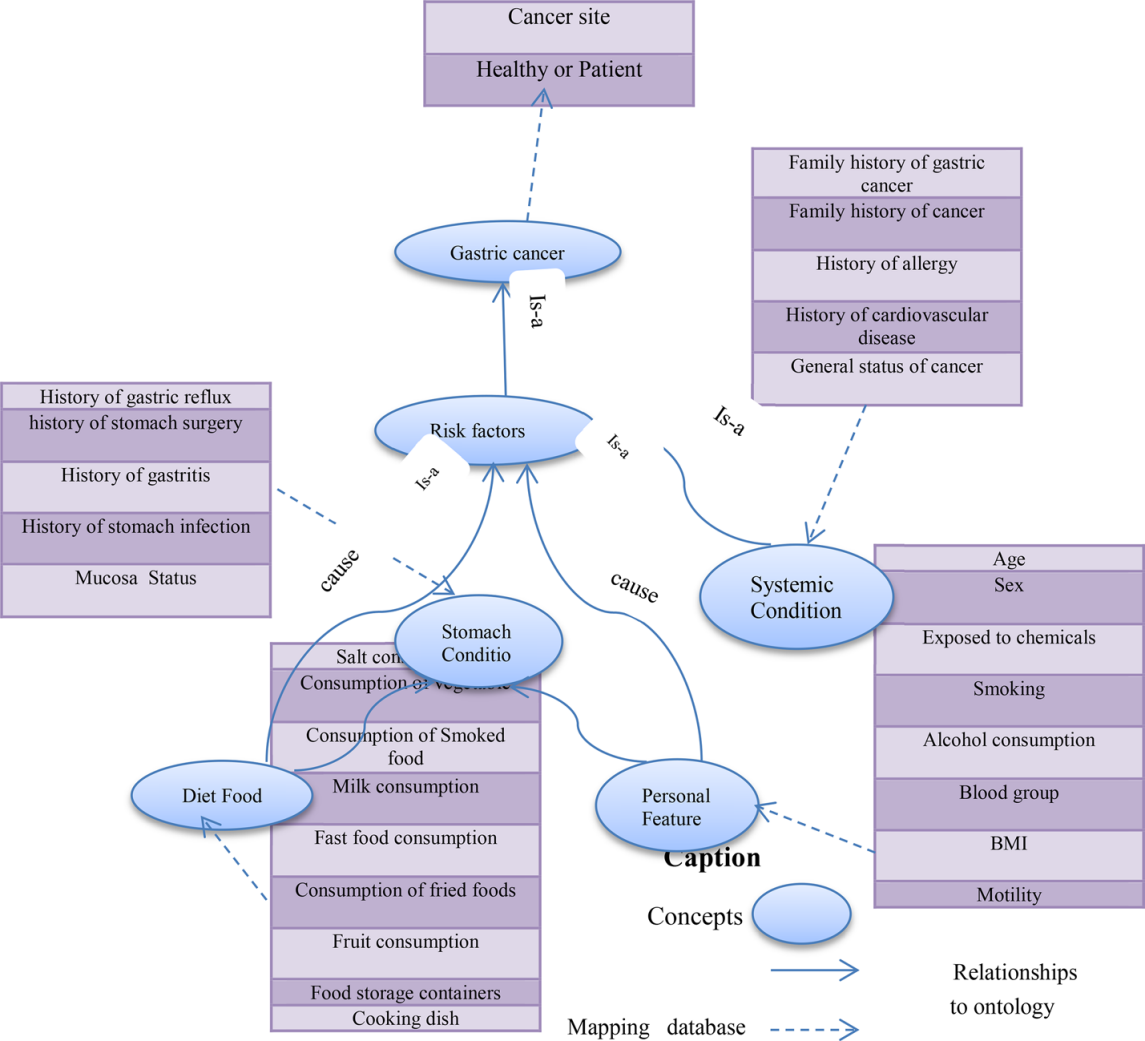
Concepts and relationships of the ontologies

As mentioned in the previous section, risk factors of gastric cancer are divided into four subclasses including stomach condition, personal feature, systemic condition, and diet food. In fact, four main concepts on risk factors of gastric cancer were introduced. All attributes available in the database mapping to concepts exist in the ontology and risk factors of cancer. The concepts and relationships of the ontologies used in this work can be seen in Fig. 6.

### Proposed algorithm

As stated, earlier studies used traditional algorithms such as Apriori to extract association rules. Disadvantages of this algorithm include:

1. The major problem of applying traditional framework of discovering association rules through using Apriori algorithm is the large numbers of rules produced. If the produced association rules were high in numbers, it would be difficult to examine by human being. Thus, it requires proper organizing to better understand and use the rules.
2. The second problem of conventional method is producing high numbers of unattractive, meaningless rules.
3. The other weakness of Apriori algorithm is its long running time. If the existing features of database under consideration are in large numbers, running the algorithm requires high cost and time.

**Fig. 7:**
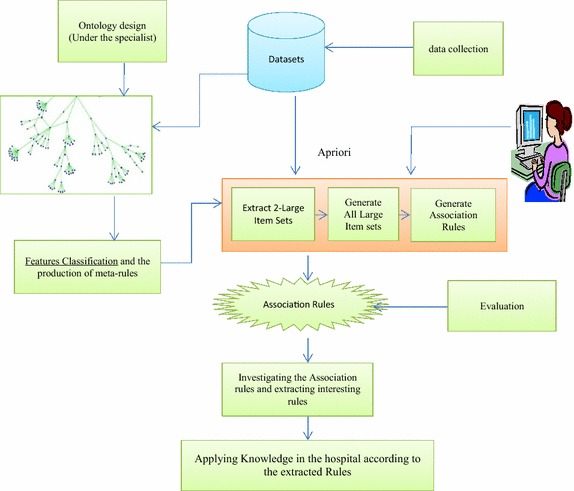
Workflow of the proposed algorithm

**Fig. 8:**
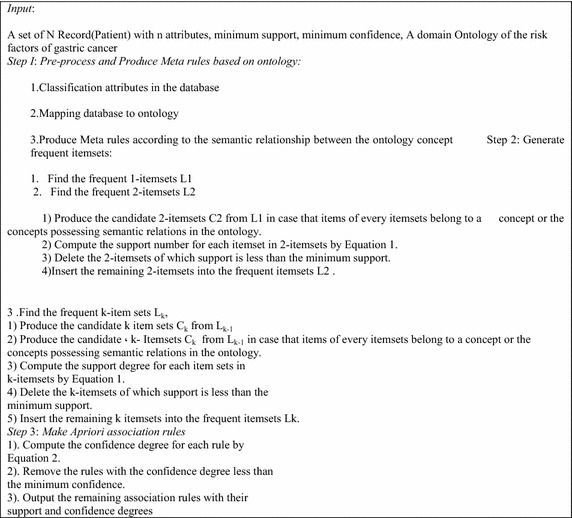
Algorithm process

Figure 7 shows the workflow of the proposed algorithm. The suggested ontology combines domain knowledge, expert knowledge, and user knowledge from data set and association rules query is performed according to anthology manual. The existing knowledge in ontology indicates specific semantic relations among classes and typically features. Semantic relations of ontology notions referred as meta Rules. Semantic content extracted from anthologies brings more knowledge and information into data mining; further, enhances the quality. Regarding suggested ontology and the existing knowledge, the created rules get more inclusive removing the difficulties of the previous method. Recommended algorithm is shown in details in Fig. 8.

#### Example

**Fig. 9:**
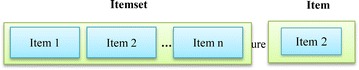
Itemset, item structure

In this example, ten patients are selected with five features and algorithms process is run on the data sets. It is noteworthy that this data is only a small part of the original data collection. Therefore, the algorithm is given in the form of an example for better understanding. Item and Itemset are defined based on Fig. 9. The dataset used in this example is shown in Table 2. In addition, it is assumed that all pre-processing steps, ontology construction and features categories are performed like the previous sections. The support and confidence are considered by 30 and 80 %, respectively.

**Table 2:** The dataset used in this example

S. no.	Gastric cancer	History of stomach surgery	Milk consumption	Smoking	Family history of gastric cancer
1	✓	✓	✗	✓	✓
2	✓	✓	✗	✓	✓
3	✓	✗	✗	✓	✓
4	✓	✓	✓	✗	✗
5	✓	✓	✓	✓	✓
6	✓	✓	✗	✓	✓
7	✗	✗	✓	✗	✓
8	✗	✗	✓	✗	✗
9	✗	✗	✓	✗	✗
10	✗	✗	✗	✓	✗

The algorithm produces the L1 elements and all the features are placed in this set based on the degree of the support. Then, the algorithm attempts to produce frequent elements with the length 2. Table 3 shows 2-Large Itemset.

**Table 3:**
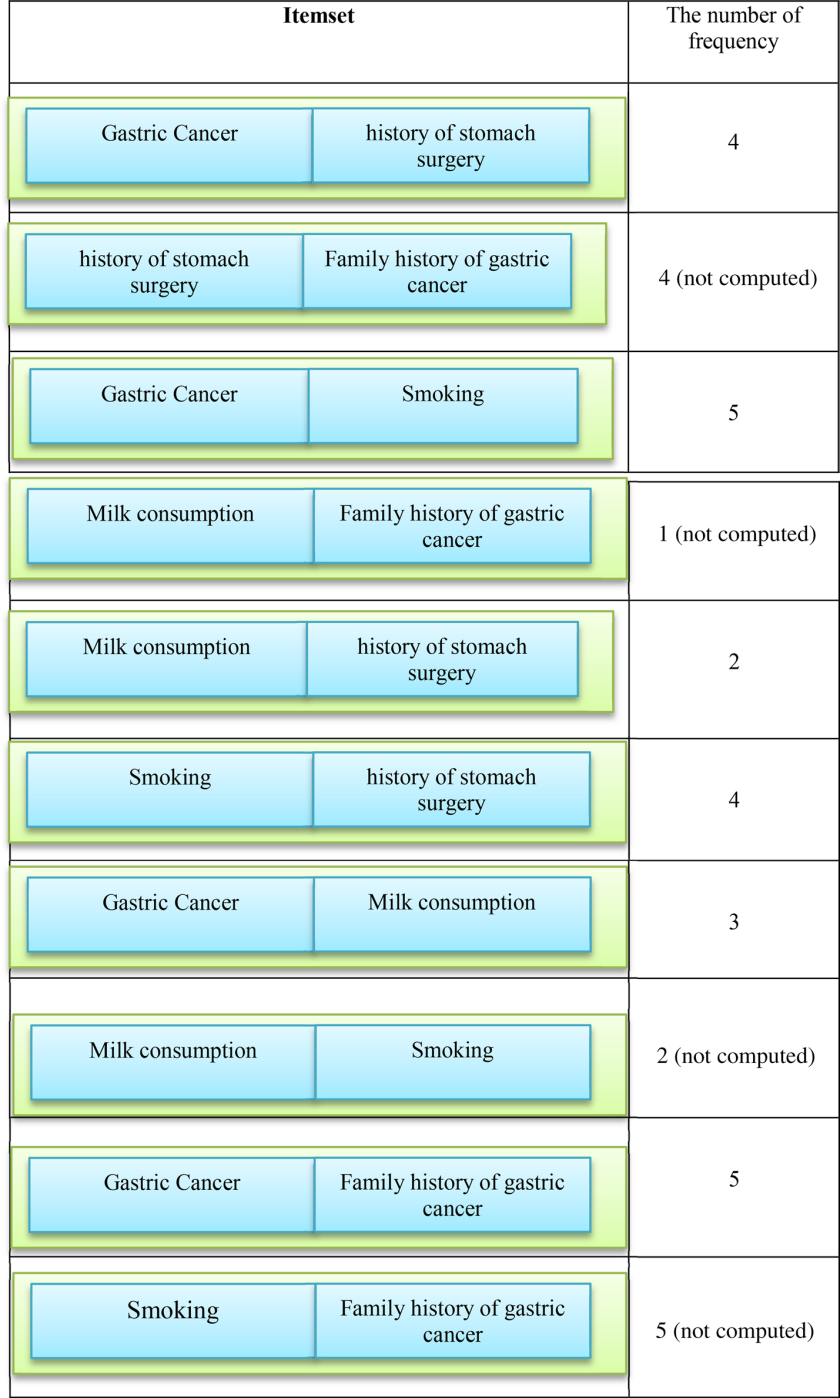
2-large itemset

After determining 2-Large Item set, items which have no semantic relationship based on concepts in the ontology and meta-rules are removed. Three semantic relationships are defined in the proposed ontology. Direct relationship is the first relationship for the ontology concepts. In this type, concepts are directly related with each other, for example, all of IS–A and Cause relationships. Indirect relationship is the second type of the relationship between the concepts in the ontology. In this case, for example, two concepts are related with each other due to the existence of another concept, for example, the concept of diet with gastric cancer is associated with each other due to the presence of risk factors. The third type of relationship is the cohort relationship. This means that all the features in a category or a concept are placed in ontology. For example, all the features in the category of systemic conditions are associated with each other and the number of their repetitions in the algorithm is computed together. According to the mentioned explanations, Item sets of {(family history of gastric cancer, gastric surgery)}, {(family history of gastric cancer, consumption of milk)}, {(smoking, consumption of milk)} {(family history of gastric cancer, smoking)} are removed because there is no semantic relationship between the items.

Then, the degree of the remaining Item sets support is computed. Table 4 shows the remaining Item sets with their support degree. Given the degree of the support, Item set with <30 % for the degree of the support is removed. In other words, the item set {(family history of gastric cancer, consumption of milk)} is removed and the remains are placed in set L2. L2 = {(A history of gastric surgery, being patient), (smoking, being patient), (history of gastric surgery, smoking) (consumption of milk, being patient), (history of gastric cancer in the family, being patient)}.

**Table 4:**
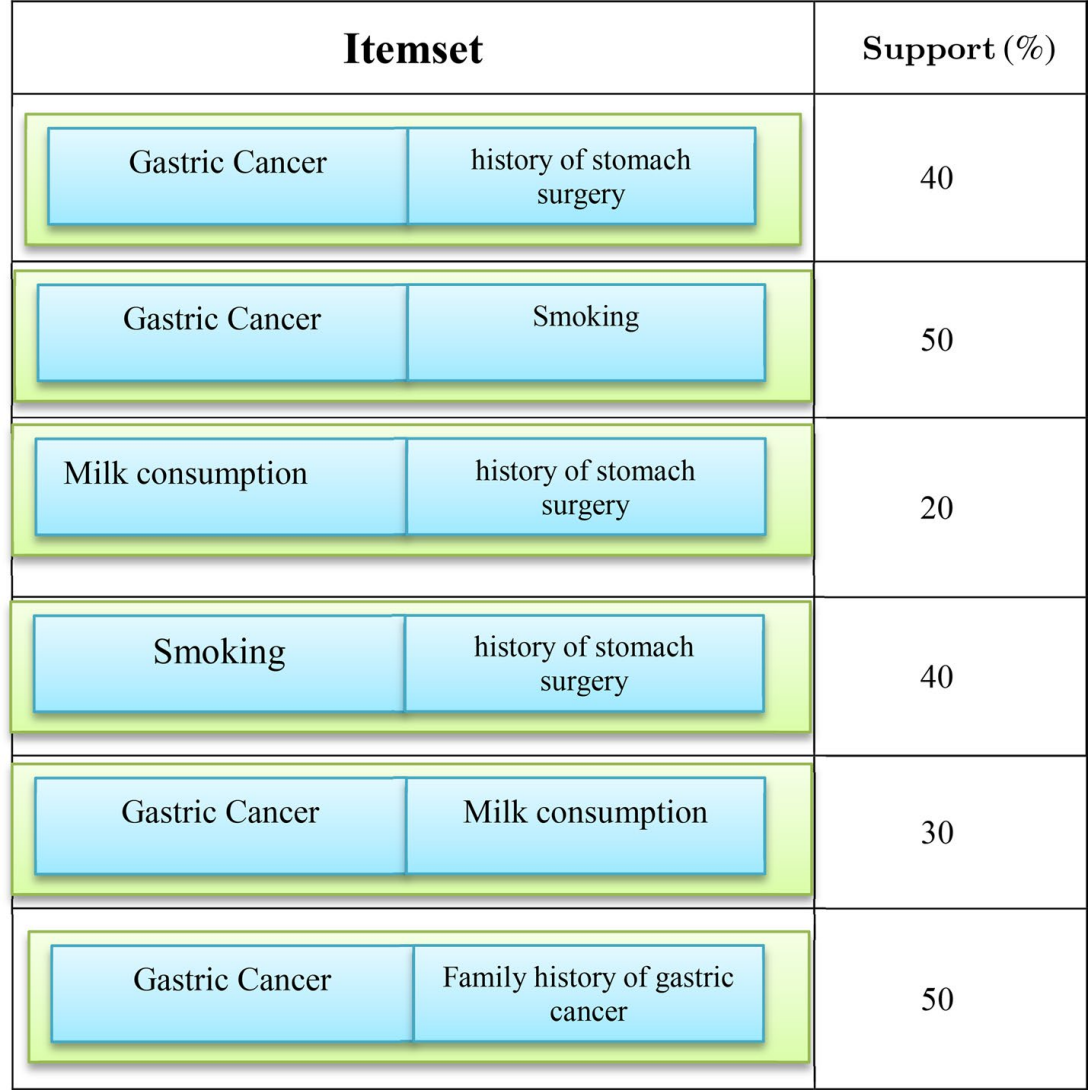
Selective itemset

In order to build a large item set with the length 3, 2-Itemsets in the previous step are combined with each individual member. Table 5 shows candidate Item sets. Before computing the Support for Item sets, Item sets whose elements have with no semantic relationship with each other are removed and only 3 3-Itemsets remain. Then, the degree of the support for the remains is computed and the degree of support less than the degree of minimum will be removed. According to Table 6, Item sets {(consumption of milk), (gastric surgery), (being patient)} will be removed. Table 7 shows the entire set of the produced rules. Table 8 shows the set of produced rules based on the confidence.

**Table 5:**
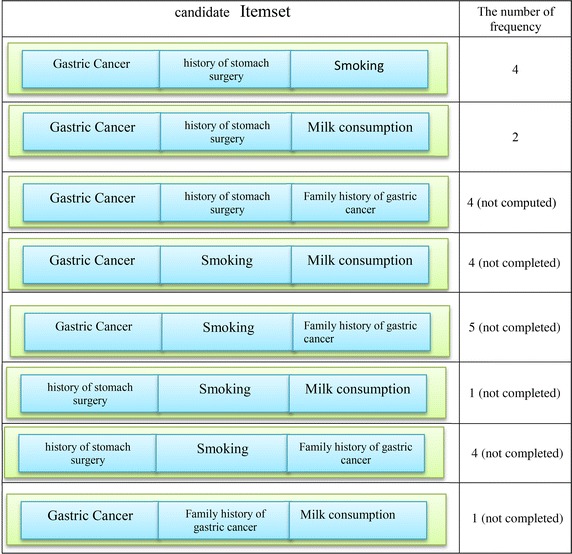
Candidate itemsets

**Table 6:**
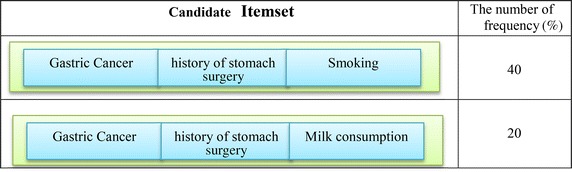
The remaining candidates itemset

**Table 7:**
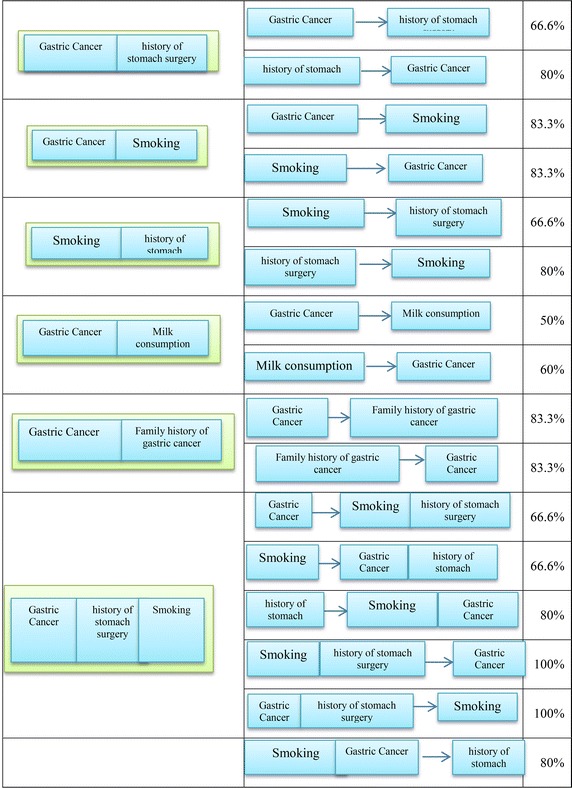
Set of production rules

**Table 8:**
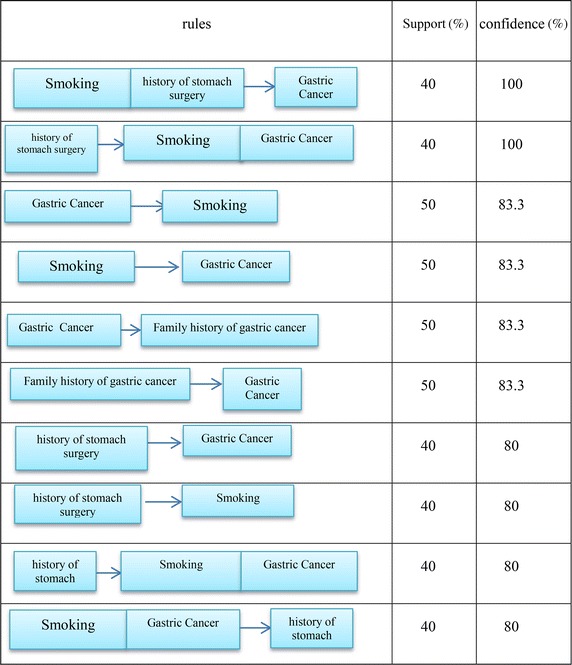
The final set of association rules

## Results and evaluation

The rules produced by the proposed algorithm are given below in decreasing support order. Because confidence is more important than support, for extracting rules the minimum acceptable confidence and support values, confidence = 1 and support = 0.2, were considered for pruning the rules. Among created rules, 18 of them that had confidence = 1 were selected. Table 9 shows the best extracted rules along with their support values that the proposed algorithm has discovered from the S-Abbas Mahmoodi dataset.

**Table 9:** The best extracted rulesrules

Rules	Result	Support
Cancer site_cardia, history of gastric reflux_yes, history of stomach infection_no	Patient	0.20612
Cancer site_cardia, history of cardiovascular disease_no, family history of cancer_no	Patient	0.21020
Cancer site_non cardia, history of gastric reflux_yes, history of stomach infection_no	Patient	0.17755
Cancer site_non cardia, history of cardiovascular disease_no, family history of cancer_no	Patient	0.18775
Cancer site_cardia, history of cardiovascular disease_no, family history of cancer_no, family history of gastric cancer_no	Patient	0.17755
Cancer site_non cardia, history of cardiovascular disease_no, family history of cancer_no, family history of gastric cancer_no	Patient	0.18775
Cancer site_cardia, History of gastric reflux_yes	Patient	0.2224
Cancer site_cardia, history of cardiovascular disease_no	Patient	0.2020
Cancer site_cardia, history of stomach infection_no	Patient	0.20612
Cancer site_non cardia, history of gastric reflux_yes	Patient	0.2244
Cancer site_non cardia, history of cardiovascular disease_no	Patient	0.2142
Cancer site_non cardia, history of stomach infection_no	Patient	0.21224
30 > BMI	History of gastric reflux_yes	0.17755
Salt consumption_not eat	History of gastric reflux_yes	0.17755
Salt consumption_high	History of gastric reflux_yes	0.20612
Milk consumption_no	History of gastric reflux_yes	0.2224
Sex_male, mucosa status_normal, exposed to chemicals_no, history of gastritis_no, alcohol consumption_no, 29.5 < BMI > 25	History of gastric reflux_yes	0.17346
Mucosa status_normal, alcohol consumption_no, motility_medium, age >61, history of stomach infection_no	History of gastric reflux_yes	0.18367

The role of ontology is important in knowledge-based systems, evaluation, and how the quality of anthology is examined. Normally, if anthologies supposed to be used, it requires some method to qualitatively measure and verify their validity. However, at present, there is no standard method for evaluating the ontology (Buitelar and Cimiano 2008) and it is regarded as a promising research area. As earlier stated, the suggested anthology designed by the experts naming pathologists.

**Fig. 10:**
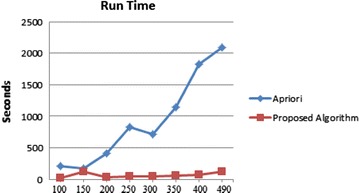
Run time

Improving Apriori algorithm by the proposed ontology, the running time of the proposed algorithm significantly reduced far more than the Apriori algorithm. To compare run time of the suggested method with Apriori algorithm, runtime of both algorithms was executed with various numbers of samples at confidence level of 0.9 and supporting level of 0.7. The results are shown in Table 10 and Fig. 10.

**Table 10:** Run time comparison

Records	100	150	200	250	300	350	400	490
Apriori	206.1	168	416.62	823.58	715.4	1150.8	1829.55	2089.46
Proposed algorithm	16.26	118.99	31.7	44.5	50.5	56.2	74.58	125.04

As Fig. 8 illustrates, the recommended algorithm runtime significantly reduced.

The major disadvantage of Apriori algorithm is producing large volume of rules. Thus, it is difficult to extract useful information from an extensive range of rules. One of the key objectives of the present study is to reduce the number of produced rules by non-production of pointless and useless rules. For this purpose, the experiments performed in terms of different supporting and confidence levels. The results are seen in Tables 11 and 12.

**Table 11:** The number of rules generated

The number of rules generated by proposed algorithm	The number of rules generated by Apriori algorithm	Minimum support	Minimum confidence
1	2	0.9	1
2	33	0.8	1
30	64	0.9	0.9
315	1422	0.8	0.9
648	8342	0.7	0.9

**Table 12:** Effect of proposed algorithm on the number of extracted rules

Percent of rules removed to total rules	Number of eliminated rules	The number of rules generated by proposed algorithm	The number of rules generated by Apriori algorithm	Minimum support	Minimum confidence
77.8	1107	315	1422	0.8	0.9
92.2	7694	648	8342	0.7	0.9

As seen, for instance, for confidence level of 0.9 and support level of 0.9, the numbers of rules resulted from running Apriori algorithm and the numbers of rules produced by running the suggested algorithm were 64 and 30 rules, respectively, which indicates decreasing numbers of rules and deleting meaningless rules.

In Table 12, confidence level is represented by first column; the second column shows support factor; the numbers of association rules resulted from Apriori algorithm and the numbers of rules produced by running the recommended algorithm are represented through third and Fourth columns, respectively; further, column five illustrates the numbers of redundant and deleted rules by the recommended method; and finally, the ratio of deleted rules to the total produced rules are seen in the sixth column.

As seen, the recommended method influenced the data used in this research consisting of 490 samples and 29 features. For instance, the number of rules produced by Apriori algorithm, at confidence and support level of 0.9 and 0.7, respectively, was 8342 rules; whereas, the rules produced by the suggested method were 648 demonstrating the 92.2 % decrease. In fact, producing 7693 meaningless and useless rules was stopped. Statistical information of the obtained results of the proposed method on dataset of the patients referring to Imam Reza hospital is represented in Table 13.

**Table 13:** Statistics obtained from the proposed algorithm

The number of rules generated	The number of large and frequent	Number of 2-large item sets	Minimum support
2635	527	92	0.5
1699	341	62	0.55
1486	260	46	0.6
1129	213	44	0.65
648	144	37	0.7
434	90	25	0.75
315	68	19	0.8
130	43	17	0.85
30	19	9	0.9

Table 13 shows the results relative to confidence level of 0.7 and support factor of 0.5–0.9 so that the algorithm behaviour is identified once support factor changes. The column “2-Large Item set” reveals that how many sets of large elements with length of 2 produced per different support values. Recall that “2-Large Item set” is the basis of creating larger elements set. The column “number of frequent and large elements” in Table 13 shows that this number depends on the number of “large element set at length 2” produced. Finally, the column “the number of produced rules” in Table 13 also represents the number of produced association rules in terms of different support factors.

## Discussion and conclusion

In this study introduced a novel approach to Extraction of Hidden Rules from gastric Cancer Data based on Ontology. Today, anthologies increasingly and widely used in knowledge engineering, artificial intelligence, computer sciences, knowledge management, natural language processing, E-commerce, information retrieval, and semantic web. Existence of languages, methods, and many tools to work on anthologies demonstrates the variety of its functional domain. The primary goal of anthology is to share knowledge among information systems; therefore, using anthology to share the knowledge in aforementioned functions is necessary (Ehrig 2007). In artificial intelligence, there are several definitions of what and how an anthology is and what its features are. The most common of the introduced definitions is Gruber’s, ontology is a conceptualization of a specification (Gruber 1993) This study provides much tangible definition of anthology as representing a particular domain in which the notions and their relations clearly and obviously indicated for a particular purpose, rather than using the aforementioned common definition.

This paper presents a mixture of Apriori Algorithm and Ontology of the risk factors of gastric cancer. In all works and studies conducted, indeed, conventional data mining was based on the frequency and iterating event occurrences and concurrency of items in transactions. The meanings of items were ignored.

The results of the experiments obtained with databases show that the suggested model fully met the desired goals. Table 9 shows the best extracting rules by using the proposed algorithm. The results reveal that:

The history of reflux highly influences the incidence. The recent findings (Carr et al. 2013) also emphasized on this. Stomach cancer is not related to cancer and stomach cancer history in family, as well as infection. Cardiovascular patients are less susceptible to stomach cancer. It worth notifying that recent studies (Kumar et al. 2013) demonstrate that cardiovascular patients are in less risk to stomach cancer due to taking some particular medications. The reflux also related to taking inadequate salt, high salt consumption, and inadequate milk taking, which can be critical issues of future studies in identifying reflux risk factors.

The association rule mining is an active research area of data mining. It is proposed and developed a new association rule method to improve the efficiency of the classic association rule mining problem, providing the ability to deal with the relationship between the domain ontology. The combination of the concept of the ontology with the standard rules mining algorithm is effective data mining approach.

For future work, it is recommended that the larger data set is used to achieve better results and more attractive rules and more comprehensive ontology is used to cover more areas of the research.
